# Spinal cord compression by a solitary metastasis from a low grade leydig cell tumour: a case report and review of the literature

**DOI:** 10.1186/1477-7819-6-75

**Published:** 2008-07-10

**Authors:** Efthimios P Samoladas, Ashraf S Anbar, Jonathan D Lucas, Hlias Fotiadis, Byron E Chalidis

**Affiliations:** 1 Spinal Unit, Guy's Hospital, London, UK; 2 Department of Orthopaedics, Veria General Hospital, Greece; 3 Department of orthopaedics, USCF Hospital, San Francisco, USA

## Abstract

**Background:**

Leydig tumour is rare and there are only three cases with metastatic disease reported.

**Case presentation:**

A 52 year-old Caucasian male was admitted, on emergency basis to the Orthopaedic Department with six weeks history of increasing midthoracic back pain, change in gait, poor balance, subjective weakness and numbness of the lower trunk and legs. MRI scan showed change in the signal intensity of T4 and T5 vertebral body but their height were maintained. Urgent T4 and T5 corpectomies, decompression of the spinal cord and reconstruction of the vertebral bodies were performed followed by radiotherapy. Neurological status significantly improved with a mild residual numbness over the dorsum of the right foot. The histology of the excised tumour was identical to the primary. At 2 years follow-up visit the patient is neurologically stable and disease free without other organs metastases.

**Conclusion:**

This is the first case in English literature, which shows that spinal metastases could occur even in the early stage of Leydig cell tumour, without other organs involvement. Aggressive surgical management of spinal metastases combined with post operative radiotherapy can give a better chance for long survivorship.

## Background

Secondary tumours are the most common tumours involving the spine [1] and their incidence may be increased as further advances in cancer therapy prolong the life expectancy of afflicted patients [2]. Malignant primary tumours most frequently metastasizing to the spine are: bronchogenic carcinoma, breast carcinoma, prostatic adenocarcinoma, renal cell carcinoma, thyroid carcinomas and GIT adenocarcinomas. Among those, metastases from the first 3 tumours are the commonest [1,3].

Leydig cell (interstitial cell) tumour of the testis was first described by Sacchi [2] in 1895. The interstitial cells of the testis, located between the seminiferrous tubules are designated by the surname of the German anatomist who first described them, Franz von Leydig. They primarily secrete testosterone [4] and it is an exceedingly rare tumour [2].

Only 7–10% of Leydig cell tumours shows malignant activity exclusively in adults [4-7] and metastasise. Moreover, it seldom metastasizes to the spine [8-10].

The tumour is generally refractory to radiotherapy and chemotherapy. The natural course of patients with metastatic variety of Leydig cell tumour is one of progression at an unpredictable pace. The median survival of these patients with metastatic disease is less than 2 years [4,11-15].

We present the fourth case in English literature of malignant Leydig cell tumour with spinal metastases and the first in the early stage of the disease. Surgical treatment in combination with post-operative radiotherapy resulted in a very satisfactory outcome. This is the first case reported with such a long disease free period.

## Case presentation

A 52 year-old Caucasian male was admitted, on emergency basis to the orthopaedic department with six weeks history of increasing mid thoracic pain, change in gait, poor balance, subjective weakness, numbness of the lower trunk and legs. He didn't report any neurogenic bladder or bowel disturbances and he was otherwise fit and well.

The patient had a right sided orchidectomy 3 years ago, for stage one well differentiated Leydig cell tumour. He was diagnosed having an enlarged right testis. No adjuvant therapy was given perioperatively. Afterwards, he followed up periodically and Computer Tomography (CT) scans of the chest, abdomen and pelvis were performed on the basis of evaluation and potential metastasizing of the neoplasm.

Two years following the primary operation the patient complained of back pain. Plain films of the spine showed an "ivory" vertebra at T4. CT scan depicted a definite abnormality in the body of T4 with no evidence of general metastatic disease. There was no soft tissue extension and no vertebral body collapse. None of the visceral organs was involved and this was the only detectable pathological sign. The bones scan showed intense uptake in T4 and no other sights of increasing radioisotope uptake. The blood tests, including tumour markers, didn't show any abnormality. At that stage, the oncologists decided against biopsy as they felt it was potentially hazardous and the patient will have little to gain from it. Accordingly, in absence of symptoms and tenderness, a "wait and see" policy was adopted.

Nine months later the patient admitted to the Spinal Unit in an emergency base complaining of increasing mid thoracic pain, change in gait, poor balance, subjective weakness, numbness of the lower trunk and legs. Examination revealed a broad base gait, able to walk in toes and heel, absence of tenderness or masses over T4 level, hypo aesthesia below T5 level more pronounced over the left side, exaggerated tendon reflexes in the lower limbs, un-sustained ankle clonus bilaterally and normal plantar reflexes. No objective motor weakness detected and intact perianal sensations were recorded.

**Figure 1:**
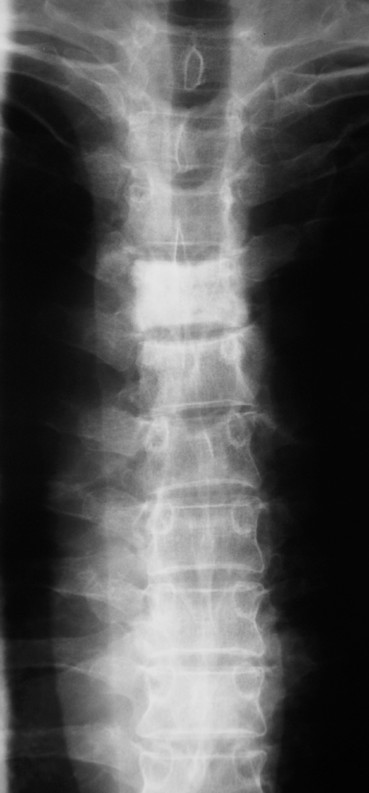
AP X ray of Thoracic spine.

**Figure 2:**
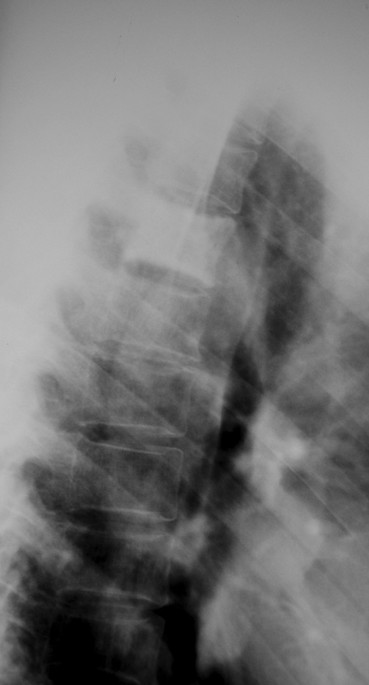
Lateral X ray of Thoracic spine.

**Figure 3:**
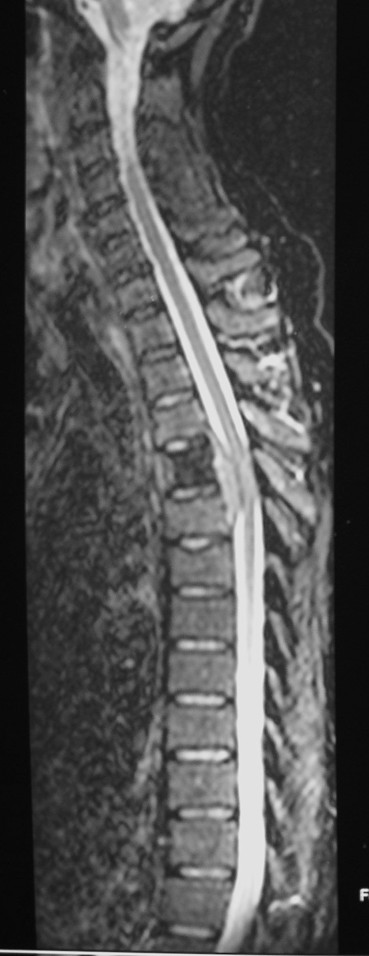
T2W MRI of Thoracic spine.

X-rays of the thoracic spine revealed a sclerotic appearance of the T4 vertebral body (Figure 1 &2) and an urgent Magnetic Resonance Imaging (MRI) showed quite dramatic change in the appearance of T4 compare to the previous CT despite the maintenance of vertebral body height. Furthermore, T5 vertebral body was also involved, but to a lesser extent. There was a soft tissue expansion into the extradural space causing spinal cord compression (Figure 3).

Blood test, including inflammatory and tumour markers, were within normal values and a dose of 16 mg Dexamethasone daily was started. A CT guided biopsy was performed and the histological appearance of the lesion was identical to the primary tumour.

After discussion with the patient, a decision was made to perform urgent T4 and T5 corpectomies, decompression of the spinal cord and reconstruction of the vertebral bodies. Anterior surgery was contemplated as the compression was coming only from the front and the tension band of the posterior elements, at the involved level, were intact.

**Figure 4:**
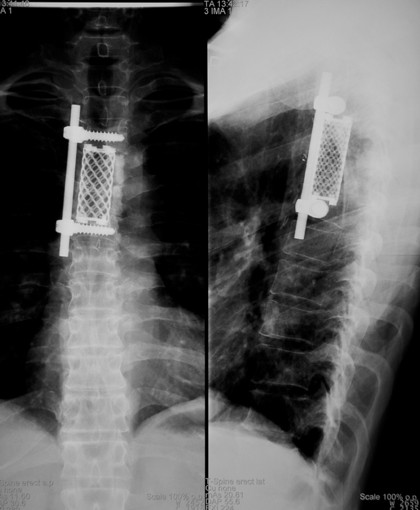
post op AP & Lat X rays of Thoracic spine.

Surgery was performed through a right subscapular 3^rd ^rib thoracotomy, and the cord function was monitored by Somatosensory Evoked Potentials (SSEPs) throughout the procedure. After complete canal decompression, reconstruction was achieved by a Synmesh packed with bone graft obtained from the excised rib. Anterior Universal Spine System (USS) II system was added to augment the construct (Figure 4).

The patient was then transferred to Intensive Therapy Unit (ITU) and had an uneventful recovery. He discharged one week following operation with clear neurological improvement. Three weeks postoperatively, he developed a right-sided pneumonia, which resolved with antibiotics. The histological evaluation of the excised tumour was identical to the primary.

Because it was a solitary metastasis and eradication of the tumour wasn't feasible by surgical excision alone as it had already extended beyond the bony limits, postoperative radiotherapy with radical intent was applied. The duration of the radiotherapy was a daily 5 weeks course and the dose was 50 Gy in 25 fractions to the area of T4/T5. Neither side effect from radiotherapy nor skin reactions was reported.

The patient was followed up periodically by Technetium bone scans and CT scans of chest, abdomen and pelvis. At the last follow-up visit-two years and six months postoperatively he was disease-free based according to the results of repeated scans. He complained only for a minimal numbness of the right foot and slight winging of the right scapula.

## Discussion

The most common sites of metastatic involvement in Leydig cell tumour are the regional lymph nodes and then the lung, liver, and bone. The spine is very rarely involved and there are only three cases reported in the English literature with spine involvement [4,5,12]. In the reported cases, spinal involvement occurred late in the course of the disease and other organs metastases had already occurred. Neurological deficit developed only as a pre-terminal event and the thoracic spine was involved in all three patients. In our case the spine was the first metastatic area without any other detectable metastases, which hasn't described before.

Traditionally, spinal metastases treatment involves radiation therapy, either alone or in conjunction with chemotherapy and/or surgical decompression. "Prophylactic" irradiation had not prevented local recurrence or metastatic spread within the radiation ports [15]. Several chemotherapeutic agents have been used in the treatment of metastatic Leydig cell tumour, with uniformly poor results [4,5,12]. Recently [16], a randomised study showed that direct decompressive surgery plus postoperative radiotherapy is superior to treatment with radiotherapy alone for patients with spinal cord compression caused by metastatic cancer.

In the reported three previous cases none treated operatively. One patient received spinal irradiation (2000 cGy) without improvement in neurological deficit but with some amelioration of back pain [4]. The second one received Mitotane(1,1-dichloro-2 [*o*-chlorophenyl]-2-[*p*-chlorophenyl]ethane or *o,p'*-DDD) chemotherapy without clear benefit [5]. The third patient received no therapy and developed progressive neurological dysfunction [12].

A combination of operative treatment and radiotherapy was adopted in our case with a satisfactory result, although the diagnosis of the metastatic disease was made at earlier stage. Our patient remained disease free at the last follow up visit two years and six months postoperatively.

It is well recognised that decompression surgery alone, whether anterior or posterior, might actually contribute to mechanical instability of the spine. This can lead to the spinal cord compression by creating post surgical deformity. Therefore, we believe that reconstruction should always be added. There is no consensus on whether stabilisation should be performed through an anterior or posterior approach since deformity and instability can be improved by either. It is frequently stated that anterior procedures give better results, but this is probably a function of patient selection [17]. If the posterior elements are not involved by the tumour, it is recommended to avoid disrupting the remaining intact posterior tension band.

However, if the patient's general condition can't allow an anterior approach, posterior decompression should always be augmented by at least posterior internal fixation and reconstruction of the anterior column also, via a lateral extracavitary approach (LECA). In our case, as the compression was mainly at the front an anterior approach with decompression and reconstruction was selected.

## Conclusion

Leydig cell tumour is a rare entity with only three reported cases of spinal metastases. They could occur even in the early stage without other organs involvement. Aggressive surgical management of spinal metastases combined with postoperative radiotherapy can give a better chance for long survivorship. Surgical planning should take into consideration that the avoidance of spinal destabilisation and the restoration of normal spinal stability are very important for the improvement of the overall outcome and quality of life.

## Competing interests

The authors declare that they have no competing interests.

## Authors' contributions

JL and ES concept and design, review of manuscript. AA helped in preparation of manuscript. HF and BC reviewed the literature and prepared the manuscript. All authors read and approved final manuscript.
